# An analysis of FRE @ BC8 SympTEMIST track: named entity recognition

**DOI:** 10.1093/database/baae101

**Published:** 2024-09-16

**Authors:** Ander Martinez, Nuria García-Santa

**Affiliations:** AI & Computing Research Group, Fujitsu Research of Europe Ltd, Camino del Cerro de los Gamos, 1, Pozuelo de Alarcón, Madrid 28224, Spain; AI & Computing Research Group, Fujitsu Research of Europe Ltd, Camino del Cerro de los Gamos, 1, Pozuelo de Alarcón, Madrid 28224, Spain

## Abstract

This paper is a more in-depth analysis of the approaches used in our submission (Martínez A, García-Santa N. (2023) FRE @ BC8 SympTEMIST track: Named Entity Recognition Zenodo.) to the ‘SympTEMIST’ Named Entity Recognition (NER) shared subtask at ‘BioCreative 2023’. We participated on the challenge submitting two systems based on a RoBERTa architecture LLM trained on Spanish-language clinical data available at ‘HuggingFace’ model repository. Before choosing the systems that would be submitted, we tried different combinations of the techniques described here: Conditional Random Fields and Byte-Pair Encoding dropout. In the second system we also included Sub-Subword feature based embeddings (SSW). The test set used in the challenge has now been released (López SL, Sánchez LG, Farré E et al. (2024) SympTEMIST Corpus: Gold Standard annotations for clinical symptoms, signs and findings information extraction. Zenodo), allowing us to analyze more in depth our methods, as well as measuring the impact of introducing data from CARMEN-I (Lima-López S, Farré-Maduell E, Krallinger M. (2023) CARMEN-I: Clinical Entities Annotation Guidelines in Spanish. Zenodo) corpus. Our experiments show the moderate effect of using the Sub-Subword feature based embeddings and the impact of including the symptom NER data from the CARMEN-I dataset.

**Database URL**: https://physionet.org/content/carmen-i/1.0/

## Introduction

Named Entity Recognition (NER) is one of the cornerstones of text mining. It is particularly useful when applied to the clinical context where Electronic Health Record (EHR) often consists of many unstructured clinical notes containing entities, such as diseases, procedures, drugs, and symptoms. The case for symptoms is particularly challenging since a single symptom can be written in many ways with varying degrees of detail. NER is necessary to go from unstructured information to structured information to perform downstream tasks. The performance of the downstream task directly depends on the performance of the NER task.

The SympTEMIST task at BioCreative VIII evaluation initiative was structured into three sub-tracks for symptom detection: Named Entity Recognition, Normalization and Entity Linking, and Multilingual Normalization. For these reasons mentioned in the previous paragraph, we found the symptom NER subtask of particular interest to us. The gold standard data is freely available at: https://zenodo.org/doi/10.5281/zenodo.8223653.

NER, as a classical Natural Language Processing (NLP) task, has a long history. Besides simple n-gram matching, a popular approach to NER was Hidden Markov Models [4, 5]. An improvement over Hidden Markov Model was applying Conditional Random Fields (CRFs) [6, 7]. With the popularization of Deep Learning (DL), Recurrent Neural Networks (RNNs) became popular for NER [8].

NER models are trained with hand-labelled (gold standard) data. This kind of data is costly to produce and therefore usually exists in limited amount. However, DL networks usually need substantial amounts of data to start producing satisfactory results. Because of this, large language models (LLMs), such as BERT [9], RoBERTa [10], became popular for NER. These models are trained on unlabelled data and serve as a basis for other downstream tasks. Nowadays, LLM-based solutions are among the most popular, and so was the case for the SympTEMIST challenge, as noted by the overview paper: ‘most teams used some sort of transformer-based approach’ [11].

Short before planning the experiments for this article, the version 1.0 of the CARMEN-I dataset was released (Announcement website: https://www.bsc.es/news/bsc-news/carmen-i-digitizing-covid-19-medical-records-artificial-intelligence). The CARMEN-I dataset includes a corpus of clinical records in Spanish language and labelled by experts. The labels included symptoms, as well as other key medical concepts such as diseases, procedures, medications, and species. Because of this match with our target task, we decided to also run experiments measuring the impact of the CARMEN-I Spanish-language symptom annotations on our results. Our goal was to see if mixing and matching this data (that was produced under different annotation criteria) would improve the performance of our solutions.

Our submitted systems are based on a RoBERTa architecture LLM trained on Spanish-language clinical data available at ‘HuggingFace’ model repository (Model name: ‘PlanTL-GOB-ES/roberta-base-biomedical-clinical-es’). The techniques that we used are CRFs, BPE-dropout, and Sub-Subword feature-based embeddings (SSW) for one of the systems. All these techniques will be briefly introduced in the ‘Techniques’ section.

The data used for the experiments in this article are available in Zenodo for the SympTEMIST dataset, at https://doi.org/10.5281/zenodo.8223654; and in PhysioNet for the CARMEN-I dataset, at https://doi.org/10.13026/bxrx-y344 (for credentialed users who sign the DUA).

## Techniques

This section describes the strategies used for our NER models. The first two, ‘CRF’ and ‘BPE dropout’, were used for both submissions. One of the systems used the ‘sub-subword features’ technique, while the other one did not.

### Conditional random fields

CRF can model the probability of transitioning from one output label to the next one. It does so by training an additional matrix (called transition matrix) in conjunction with the model. The ‘Viterbi’ algorithm is usually applied to consider different output sequences on prediction. In NER tasks following a schema such as BIO, Beginning-Inside-Outside [12], CRF can help avoid impossible transitions. The original BERT paper [9] demonstrated the usability of BERT for NER, but it did not use CRF. Later authors, such as [13], showed that CRF improved the results in some cases.

### BPE dropout

BPE dropout [14] can regularize NLP models by varying the way text is represented, resulting in an effect similar to data augmentation. It was introduced as an alternative to reference [15], where they found that the main drawback to the subword regularization method is its complexity since it requires training a unigram language model and it uses ‘Expectation–Maximization’ (EM) and ‘Viterbi’ [16] algorithms to sample segmentations.

One of the benefits of ‘BPE dropout’ is that it works on ‘BPE’ vocabulary models [17]. BPE is frequently used by ‘RoBERTa’, and as such, we did not need to rebuild the vocabularies. In comparison, the ‘unigram language model subword regularization’ method uses a statistical model and dynamic programming to be able to sample different segmentations from the same sequence. BPE dropout uses random noise to discard certain merge-operations, randomly generating a different sequence of subwords each time. This is so because BPE does not store the frequencies of each subword, only the order of the merge-operations. Merge-operations are discarded with a probability *p*, which is usually 0.1. Provilkov *et al*. [14] concluded through several experiments that BPE dropout achieves better results. Our systems used ‘BPE dropout’ during training, with a dropout probability *p* of 0.1.

### Sub-subword features

We used the Sub-subword feature method [18] in one of the systems to expose the character-level information to the network. According to [19], the sub-subword features method helps regularize the systems with little training data. The method consists in building the embedding matrices from the n-gram features of the subwords in the vocabulary. The features used to produce the embeddings are selected by an algorithm before training, and the neural network that produces the embeddings is trained with the rest of the model.

Since we used a RoBERTa LLM to build the NER models, we did not want to discard its (sub-)word embeddings. Before training the NER model using the sub-subword features embeddings, we fit the feature-to-embedding (FTE) network to produce embeddings similar to those included with the RoBERTa model. We used ‘Mean Squared Error’ (MSE) training for this purpose. After this step, the NER model was used normally (using CRF and BPE dropout).

This technique was originally proposed for NMT, and our participation on the SympTEMIST task was the first time that it was used for NER. The size of the FTE network was three layers of 3072 units in the hidden layers, as used by [19].

## Previous experiments

In order to choose the best approach for our submissions, we performed some experiments using the provided training data. The data provided contained 750 documents. The documents were segmented into sentences using Spanish-language NLTK ‘punkt’. We avoided splitting sentences when that would split a labelled entity. After sentence segmentation the dataset contained 12 009 sentences. Of these sentences we made a training, validation and test datasets that contained 11 009, 500 and 500 sentences, respectively.

We used BIO encoding for the entities. In preliminary experiments we did not find any benefit in using S- or E-tags. We first tried using a SoftMax layer on top of an LLM model. We tried different Spanish-language models available at ‘HuggingFace’ and finally the model by [20] gave best results for us, with 65.78% F1 score. We used BPE dropout to improve the F1 score to 72%.

We observed that our models were producing invalid transitions, such as outputting ‘I-SINTOMA’ labels without a preceding ‘B-SINTOMA’. For this reason, we decided to try using CRF on top of the LLM-based NER model, which improved the F1 score. Since our predictions were still producing invalid transitions, we initialized the CRF transition matrix to disallow O- to I-transitions. The introduction of this bias gave us the best results.

We also tried using the Sub-subword features approach described in the techniques section. This did not improve the F1 score for us.

We trained all the models for 25 epochs with batches of 15 sentences and learning rate of 2e-5. ‘AdamW’ optimizer was used, keeping the best model according to our validation data. The results of these preliminary experiments are summarized in the first column of Table 2. Unlike the other reported results, these results were computed on our custom test set, randomly partitioned from the training data.

**Table 2.** Entity-level F1 scores computed on three runs with different seeds

		roberta-base-biomedical-es			bsc-bio-es		
F1 (entity level)	Previous	Mean (SD)	Min	Max	Mean (SD)	Min	Max
Softmax	65.78%	66.25 ±0.34	65.99	66.64	65.57 ±0.41	65.28	66.04
+CARMEN-I	–	66.38 ±0.59	65.79	66.97	65.84 ±0.14	65.73	66.00
Softmax + BPEd	72.12%	68.31 ±0.46	67.91	68.81	67.84 ±0.25	67.55	67.98
+CARMEN-I	–	68.80 ±0.35	68.39	69.02	66.89 ±1.05	65.74	67.82
CRF + BPEd	75.77%	70.12 ±0.61	69.43	70.56	69.02 ±0.45	68.51	69.37
+CARMEN-I	–	71.47 ±0.68	70.80	72.15	69.45 ±0.95	68.48	70.38
CRF + BPEd + bias	78.03%	71.80 ±1.13	70.51	72.60	71.70 ±0.31	71.40	72.02
+CARMEN-I	–	72.45 ±0.46	71.94	72.86	71.91 ±0.27	71.60	72.07
CRF + BPEd + bias + SSWF	77.92%	70.06 ±0.64	69.38	70.66	69.42 ±0.90	68.53	70.34
+CARMEN-I	–	70.43 ±0.72	69.73	71.17	70.39 ±0.08	70.32	70.48

– indicates data are not available.

Since multiple submissions were allowed for each team, we submitted two systems corresponding to the *CRF + BPEd + bias* and *CRF + BPEd + bias + SSWF* from Table 2 but trained on the whole training data for a fixed number of four epochs. We decided to run for four epochs because we observed from the preliminary experiments that, for these configurations, the best performing model was usually found at epoch 4 for all initialization seeds.

We reproduced the official results for our two submissions in Table 1, together with the results of the best-performing submission for strict evaluation (an ensemble model [21] from ICB team) and for overlapping evaluation (an ensemble model [22] from BIT.UA team). The scores P and R stand for precision and recall. The scores prefixed by ‘o_’ show their overlapping counterpart. We only considered strict F1 score to optimize our models. The best scores are highlighted in bold and second-best in underline.

**Table 1.** Results reported by the organizers

Team’s name	Run name	P	R	F1	o_P	o_R	o_F1
FRE	1-roberta	0.7231	0.7303	0.7267	0.8616	0.8702	0.8658
FRE	2-roberta_ssw	0.7154	**0.7403**	0.7277	0.8487	**0.8782**	0.8632
ICB	icb-uma-ensemble	**0.8039**	0.6988	**0.7477**	**0.9155**	0.7957	0.8514
BIT.UA	1-system-all	0.7473	0.7258	0.7364	0.8816	0.8563	**0.8688**

Scores prefixed by ‘o_’ report overlapping results. The figures in bold are the highest values in their column.

Although our submissions were not among the best with respect to the F1 score they did get the best recall scores for strict and overlapping evaluation. On overlapping evaluation, our models had better F1 score than the best model from strict evaluation, which was optimized for precision. The overlapping F1 of our models was close to the best performing model from team BIT.UA.

## New experiments

A new version of the SympTEMIST dataset was released after the completion of the challenge [2]. This new version included the held-out test set and normalized data. With this new data, we repeated the experiments evaluating the results on the provided test data.

The hyperparameters for the new set of experiments were as described for the previous set. We used 1000 sentences from the training data for validation and chose the model producing the best validation. We trained this model 1 extra epoch using a combination of the training data and validation data. This is different from the four epoch approach that we took for the challenge submissions.

We observed that the models roberta-base-biomedical-es (Model name: ‘PlanTL-GOB-ES/roberta-base-biomedical-es’) and bsc-bio-es (Model name: ‘PlanTL-GOB-ES/bsc-bio-es’) were used by other participants. We compare these two pretrained LLMs. We also experimented with adding NER training data from the CARMEN-I dataset.

We report the mean and standard deviation of the F1 score, as well as the minimum and maximum scores, for each model configuration. The results are summarized in Table 2.

The trend that we observed with the previous set of experiments is repeated and each of the added techniques improves the result except for the sub-subword features, which had a negative impact on F1 scores. We also see that the results that we obtained are different from the official results. We cannot explain this difference, but it may be related to a different pre-/post-processing or differences in the evaluation code.

The roberta-base-biomedical-es (RBBE)-based models performed slightly, but consistently better than bsc-bio-es (BBE)-based ones.

Including the data from CARMEN-I generally improved the results, but just slightly in most cases. The reason for the lack of larger improvements may be the different nature of the texts in CARMEN-I.

## Model ensembling experiments

The best performing model [21] in the official ranking was an ensemble of multiple models using a simple majority voting approach. We also tried this approach using the models from Table 2. Since we trained three runs for each model configuration, we tried combining the three of them for the CRF + BPEd + bias configuration (-sswf) and CRF + BPEd + bias + SSWF configuration (+sswf).

We did these for both RBBE and BBE. These ensembles use three models. The results are displayed in Table 3. The (-sswf + sswf) row combines the models from the two previous columns, and thus, the cells (RBBE,-sswf + sswf) and (BBE,-sswf + sswf) each ensemble six models. The RBBE + BBE column is the combination of the two previous columns, so its cells use 6, 6, and 12 models, respectively. We repeated this for the models using training data from CARMEN-I corpus.

**Table 3.** Ensemble model results

	-CARMEN-I			+CARMEN-I		
	RBBE	BBE	RBBE + BBE	RBBE	BBE	RBBE + BBE
-sswf	73.02	72.64	73.98	73.47	72.59	74.21
+sswf	70.99	70.64	71.73	71.56	71.91	72.24
-sswf + sswf	73.50	72.72	73.67	73.60	73.09	74.23

RBBE = roberta-base-biomedical-es; BBE = bsc-bio-es

The ensemble models improve the results of their corresponding base models in all cases, also when all the models used the same configuration. However, we observe that the improvement is larger when the models are of different configurations.

## Conclusions

Our experiments confirmed the efficacy of well-stablished NER techniques. Our experimental SSWF technique did not behave as well as we had expected, but it did improve the results when combined with other models in an ensemble setting. Using the extra data from CARMEN-I did generally improve the result in spite of the format difference in the source text data.

## References

[1] Martínez A , García-Santa N. FRE @ BC8 SympTEMIST track: Named Entity Recognition. Zenodo, 2023. 10.1093/database/baae101 PMC11403810 39283593

[2] López SL , Sánchez LG, Farré E et al. SympTEMIST Corpus: Gold Standard annotations for clinical symptoms, signs and findings information extraction. Zenodo, 2024.

[3] Lima-López S , Farré-Maduell E, Krallinger M. CARMEN-I: clinical entities annotation guidelines in Spanish. Zenodo, 2023.

[4] Mayfield J , McNamee P, and Piatko C. Named Entity Recognition using hundreds of thousands of features. In: *Proceedings of the Seventh Conference on Natural Language Learning at HLT-NAACL 2003*, Edmonton. pp. 184–87. ACL, 2003.

[5] Morwal S , Jahan N, Chopra D. Named Entity Recognition using Hidden Markov Model (HMM). *Int J Nat Lang Comput* 2012;1:15–23. doi: 10.5121/ijnlc.2012.1402

[6] Lafferty J , McCallum A, and Pereira F. Conditional Random Fields: Probabilistic Models for Segmenting and Labeling Sequence Data. 2001. https://www.semanticscholar.org/paper/Conditional-Random-Fields%3A-Probabilistic-Models-for-Lafferty-McCallum/f4ba954b0412773d047dc41231c733de0c1f4926 (2 May 2023, date last accessed).

[7] Finkel JR , Grenager T, Manning C. Incorporating non-local information into information extraction systems by Gibbs Sampling. In: *Proceedings of the 43rd Annual Meeting of the Association for Computational Linguistics (ACL’05)*. pp. 363–70. Ann Arbor, Michigan: Association for Computational Linguistics, 2005.

[8] Chowdhury S , Dong X, Qian L et al. A multitask bi-directional RNN model for named entity recognition on Chinese electronic medical records. *BMC Bioinf* 2018;19:499. doi: 10.1186/s12859-018-2467-9 PMC6309059 30591015

[9] Devlin J , Chang MW, Lee K et al. BERT: pre-training of deep bidirectional transformers for language understanding. arXiv. 2019.

[10] Liu Y , Ott M, Goyal N et al. RoBERTa: a robustly optimized BERT pretraining approach. arXiv. 2019.

[11] Lima-López S , Farré-Maduell E, Gasco-Sánchez L et al. Overview of Symptemist at Biocreative VIII: corpus, guidelines and evaluation of systems for the detection and normalization of symptoms, signs and findings from text. In: *Proceedings of the Biocreative VIII Challenge and Workshop: Curation and Evaluation in the Era of Generative Models*, New Orleans, LA, pp. 15. Zenodo, 2023.

[12] Ramshaw LA , and Marcus MP. Text Chunking using Transformation-Based Learning. arXiv. 1995. http://arxiv.org/abs/cmp-lg/9505040 (15 May 2023, date last accessed).

[13] Souza F , Nogueira R, and Lotufo R. Portuguese Named Entity Recognition using BERT-CRF. arXiv. 2020. http://arxiv.org/abs/1909.10649 (10 May 2023, date last accessed on ).

[14] Provilkov I , Emelianenko D, and Voita E. BPE-Dropout: Simple and Effective Subword Regularization. arXiv. 2020. http://arxiv.org/abs/1910.13267 (2 May 2023, date last accessed).

[15] Kudo T . Subword regularization: improving neural network translation models with multiple subword candidates. In: *Proceedings of the 56th Annual Meeting of the Association for Computational Linguistics (Volume 1: Long Papers)*. pp. 66–75. Melbourne, Australia: Association for Computational Linguistics, 2018.

[16] Viterbi A . Error bounds for convolutional codes and an asymptotically optimum decoding algorithm. *IEEE Trans Inf Theory* 1967;13:260–69.

[17] Sennrich R , Haddow B, and Birch A. Neural Machine Translation of Rare Words with Subword Units. ArXiv150807909 Cs. 2015. http://arxiv.org/abs/1508.07909 (3 May 2023, date last accessed).

[18] Martinez A , Sudoh K, Matsumoto Y. Sub-subword N-gram features for subword-level neural machine translation. *J Nat Lang Process* 2021;28:82–103.

[19] Martinez A . The Fujitsu DMATH submissions for WMT21 news translation and biomedical translation tasks. In: *Proceedings of the Sixth Conference on Machine Translation*, Online. pp. 162–66. Online: Association for Computational Linguistics, 2021. https://arxiv.org/abs/2109.03570.

[20] Carrino CP , Armengol-Estapé J, Gutiérrez-Fandiño A et al. Biomedical and clinical language models for Spanish: on the benefits of domain-specific pretraining in a mid-resource scenario. 2021. https://arxiv.org/abs/2109.03570 (20 March 2024, date last accessed).

[21] Gallego F , Veredas FJ. ICB-UMA at BioCreative VIII @ AMIA 2023 Task 2 SYMPTEMIST (symptom text mining shared task). Zenodo, 2023.

[22] Jonker RAA , Almeida T, Matos S et al. Team BIT.UA @ BC8 SympTEMIST track: a two-step pipeline for discovering and normalizing clinical symptoms in Spanish. *Proc BioCreative VIII Chall Workshop Curation Eval Era Gener Models*, New Orleans, LA. pp. 17. Zenodo, 2023.

